# The complete chloroplast genome sequence of *Solanum hougasii*, one of the potato wild relative species

**DOI:** 10.1080/23802359.2018.1491342

**Published:** 2018-07-11

**Authors:** Kwang-Soo Cho, Ji-Hong Cho, Ju-Sung Im, Jang-Gyu Choi, Young-Eun Park, Dong-Chil Jang, Su-Young Hong, Tae-Ho Park

**Affiliations:** a Highland Agriculture Research Institute, National Institute of Crop Science, Rural Development Administration, Pyeongchang, South Korea;; b Department of Horticulture, Daegu University, Gyeongsan, South Korea

**Keywords:** Chloroplast, genome, genome sequence, *Solanum hougasii*

## Abstract

*Solanum hougasii* is a wild tuber-bearing species belonging to the family Solanaceae. The complete chloroplast genome of *S. hougasii* was constituted by *de novo* assembly, using a small amount of whole genome sequencing data. The chloroplast genome of *S. hougasii* was a circular DNA molecule with a length of 155,549 bp and consisted of 85,990 bp of large single copy, 18,373 bp of small single copy, and 25,593 bp of a pair of inverted repeat regions. A total of 158 genes were annotated, including 105 protein-coding genes, 45 tRNA genes, and eight rRNA genes. Maximum likelihood phylogenetic analysis with 25 Solanaceae species revealed that *S. hougasii* is most closely grouped with *S. tuberosum*.

*Solanum hougasii*, a wild tuber-bearing hexaploid species, is a relative to the cultivated potato, *S. tuberosum*. It was identified to be a source of resistance to late and early blight, root-knot nematode, and potato virus Y for potato breeding (Cockerham 1970; Brown et al. 1999; Inglis et al. 2007; Haynes and Qu 2016). Its EBN (Endosperm Balanced Number) value of four, theoretically makes it directly crossable for breeding purposes with cultivated tetraploid potatoes (Hawkes 1990; Ortiz and Ehlenfeldt 1992; Cho et al. 1997; Spooner et al. 2014; Haynes and Qu 2016). Moreover, its nuclear genome composition has evolutionally been identified by GISH analysis (Pendinen et al. 2012). *S. hougasii* has an allotropic behavior, that is, one genome belonged to AA and the other to BB. In addition, *S. hougasii* third genome is more intimately related to P genome or to the species related to P genome (Pendinen et al. 2012). The information of plastid genome of the wild species obtained in this study will provide an opportunity to investigate more detailed evolutionary and breeding aspects.

The *S. hougasii* (PI161174) was originally collected in Michoacan, Mexico by International Potato Centre (CIP), provided via Highland Agriculture Research Institute and stored at Daegu University, South Korea. A paired-end (PE) genomic library was constructed with total genomic DNA, according to the standard protocol (Illumina, San Diego, USA) and sequenced using an HiSeq2000 at Macrogen (http://www.macrogen.com/kor/). Low-quality bases with raw scores of 20 or less were removed and approximately 5.1 Gbp of high-quality PE reads were assembled by a CLC genome assembler (CLC Inc, Rarhus, Denmark) (Kim et al. 2015). The reference chloroplast genome sequence of *S. commersonii* (KM489054, Cho et al. 2016) was used to retrieve principal contigs representing the chloroplast genome from the total contigs using Nucmer (Kurtz et al. 2004). The representative chloroplast contigs were arranged in an order based on BLASTZ analysis (Schwartz et al. 2003) with the reference sequence and were connected to a single draft sequence by joining overlapping terminal sequences. DOGMA (Wyman et al. 2004) and BLAST searches were used to predict the chloroplast genes.

The complete chloroplast genome of *S. hougasii* (GenBank accession no. MF471372) was 155,549bp in length and included 25,593bp inverted repeat (IRa and IRb) regions separated by small single copy (SSC) region of 18,373bp and large single copy (LSC) region of 85,990bp with the typical quadripartite structure of most plastids, and the structure and gene features were typically identical to those of higher plants. A total of 158 genes with an average size of 584.5bp were annotated including 105 protein-coding genes with an average size of 766.6bp, 45 tRNA genes, and 8 rRNA genes. An overall GC content was found to be 37.87%.

Phylogenetic analysis was performed using chloroplast coding sequences of *S. hougasii* and 25 published species in Solanaceae family by a maximum likelihood method in MEGA 6.0 (Tamura et al. 2013). According to the phylogenetic tree, *S. hougasii* belonged to the same clade in *Solanum* species as expected, and was interestingly closely grouped with *S. tuberosum* (Figure 1).

**Figure 1.**
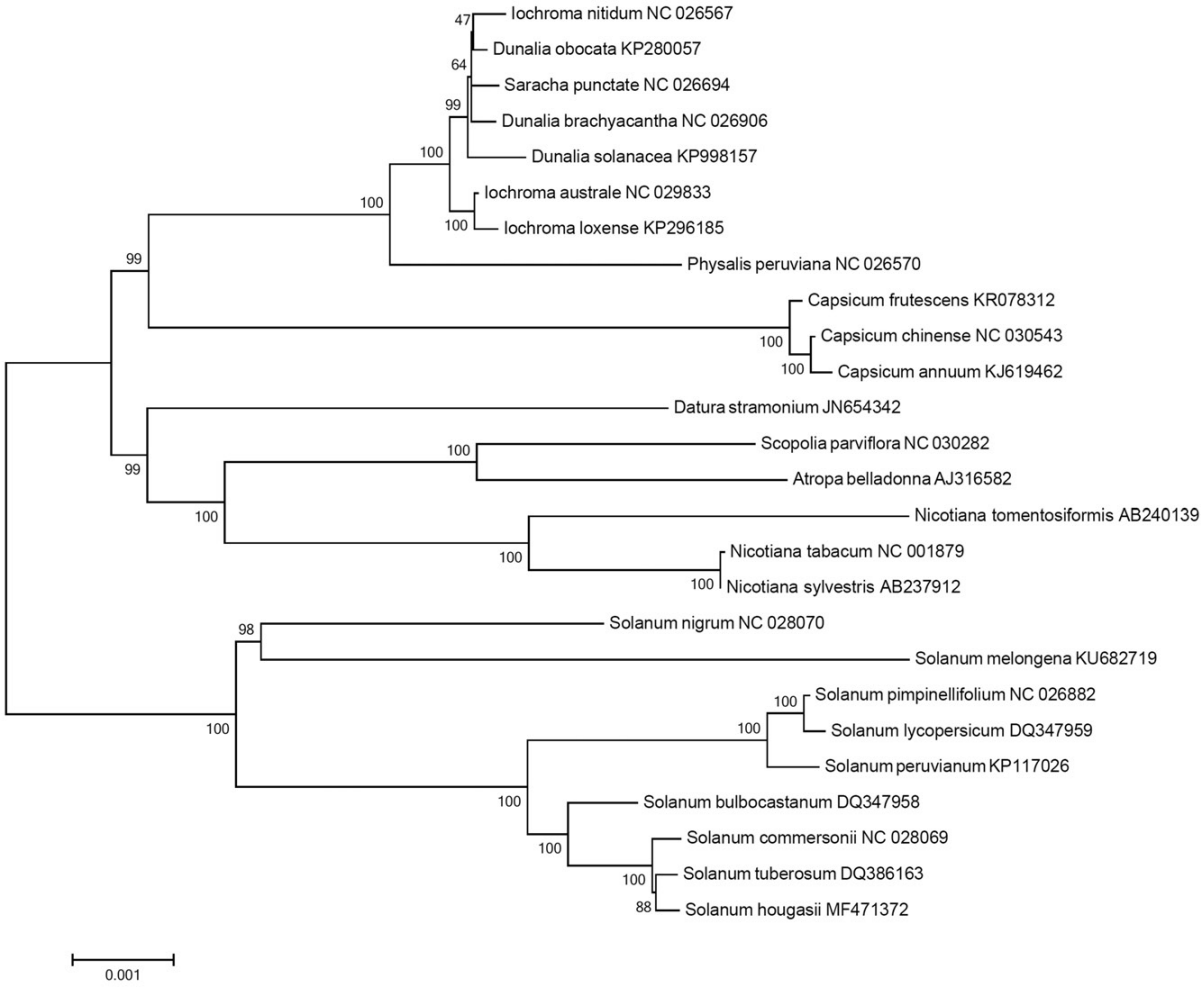
Maximum likelihood phylogenetic tree of *S. hougasii* with 25 species belonging to the Solanaceae based on chloroplast protein coding sequences. Numbers in the nodes are the bootstrap values from 1,000 replicates.
